# Cross-kingdom microbiome interactions along the gut–lung axis: immune–microecological coordination, shared mechanisms, and disease-context dependence in respiratory disorders

**DOI:** 10.1080/19490976.2026.2689168

**Published:** 2026-06-21

**Authors:** Jiang Yu, Jianbiao Meng, Zhongliang Shi, Jitao Zou, Zhizhen Lai

**Affiliations:** a Tongde Hospital of Zhejiang Province Affiliated to Zhejiang Chinese Medical University (College of Integrated Traditional Chinese and Western Medicine Clinical Medicine), Hangzhou, Zhejiang, People's Republic of China; b Department of Critical Care Medicine, Tongde Hospital of Zhejiang Province, Hangzhou, Zhejiang, People's Republic of China; c Zhejiang Academy of Traditional Chinese Medicine, Hangzhou, Zhejiang, People's Republic of China

**Keywords:** Gut microbiome, cross-kingdom interactions, gut–lung axis, mycobiome, virome, bacteriophages, lung diseases

## Abstract

Cross-kingdom dysbiosis of the gut microbiome along the gut–lung axis has emerged as a key driver of chronic and acute respiratory diseases. Beyond bacteria, the intestinal mycobiome and virome, including bacteriophages, shape mucosal immunity and metabolism through partially overlapping but non-redundant pathways. In this Review, we synthesize rapidly expanding evidence that fungi, bacteria, and phages in the gut form an integrated network that may influence susceptibility, inflammatory tone, and therapeutic responsiveness across asthma, chronic obstructive pulmonary disease (COPD), acute respiratory distress syndrome (ARDS), and lung cancer via the gut–lung axis. We first summarize how cross-kingdom communities in the intestine are organized and interact, highlighting a tripartite framework centered on pathogen-associated molecular pattern–pattern recognition receptor (PAMP–PRR) circuits, the short-chain fatty acid (SCFA)–regulatory T-cell axis, and tryptophan–indole–aryl hydrocarbon receptor (AHR) signaling. We then compare how these shared axes are differentially perturbed across asthma, COPD, ARDS, and lung cancer, using these disorders as representative but non-sequential disease contexts along a conceptual gradient of immune–microecological disruption. Finally, we discuss how dietary modulation, pre-/pro-/postbiotics, mycobiome- and virome-targeted strategies, and phage-based approaches could be rationally combined to restore gut-derived immunometabolic circuits and improve respiratory outcomes. By integrating cross-kingdom ecology with mucosal immunology, this Review provides an integrative interpretive framework suggesting that gut microbiome-targeted strategies may help refine prevention, stratification, and adjunctive treatment approaches in selected respiratory disease contexts.

## 1. Introduction

Immune–microecological coordination between distant organs has become one of the most promising research directions in respiratory and gastrointestinal medicine. The “gut–lung axis” (GLA) provides a new framework for understanding systemic inflammation, mucosal immunity, and multi-organ interactions. A growing body of basic and clinical evidence shows that the gut microbiota influences not only local mucosal homeostasis but also pulmonary immunity through microbial metabolites, cytokines, immune-cell trafficking, and neuroendocrine pathways. These processes are closely linked to the onset and progression of major respiratory diseases, including asthma, chronic obstructive pulmonary disease (COPD), acute respiratory distress syndrome (ARDS), and lung cancer.^1-4^

However, the current gut–lung axis literature remains uneven in at least three respects. First, most reviews are dominated by bacterial and metabolite-centered perspectives, whereas fungal and virome/phage components are often discussed only briefly or in isolation. Second, the mechanistic discussion is largely gut-centered, with relatively limited attention given to lung-resident microbial communities and their interaction with gut-derived immune signals. Third, many mechanistic insights are derived from murine or reductionist experimental models, whereas causal evidence from human respiratory disease remains comparatively limited. These disparities complicate translational interpretation and justify a more explicit cross-kingdom and evidence-graded synthesis.

Compared with previous gut–lung axis reviews that focused primarily on bacterial communities, microbial metabolites, or single disease settings, this Review integrates bacteria, fungi, and the virome/phageome into a cross-kingdom and evidence-graded framework. Its main contribution is to compare how shared immune–metabolic axes are differentially expressed across non-sequential respiratory disease contexts, while explicitly distinguishing mechanistic evidence, human associative findings, and hypothesis-generating interpretations. We therefore aim not to propose a new canonical pathway, but to provide a critical scaffold for identifying where gut-derived signals, lung-local microbial communities, and their interactions are supported, uncertain, or still untested.

With advances in metagenomics, virus-like particle (VLP) enrichment sequencing, and multi-omics approaches across microbial domains, increasing evidence indicates that tripartite interactions among fungi, bacteria, and viruses are not merely ecological phenomena. They represent a key regulatory layer that shapes immune homeostasis, determines inflammatory phenotypes, and affects responses to therapy. More specifically, the fungi-driven Dectin-1/CARD9–Th17 axis, the virus/phage-mediated TLR9–IFN axis and RIG-I/MDA5–type I interferon (IFN-I) axis, and the bacteria-dominated PAMP–PRR and SCFA–Treg axis together can be integrated into what we refer to in this Review as a “cross-kingdom tri-axial framework”, an author-defined organizing construct rather than an established formal model. Its purpose is not to claim three entirely novel pathways, but to provide a comparative scaffold for integrating shared immune and metabolic circuits across bacterial, fungal, and phage-mediated gut–lung interactions. This mechanism can spread between the gut and the lung, and can amplify or suppress inflammation depending on context.^5-7^

In this Review, asthma, COPD, ARDS, and lung cancer are not arranged as a linear disease continuum, nor are they intended to imply shared clinical progression. Instead, these conditions are selected as contrasting but partially intersecting respiratory settings that collectively illustrate how cross-kingdom microbiome perturbation may operate under different immunological and ecological constraints. Asthma primarily reflects early-life or type 2/17-skewed immune programming, COPD emphasizes chronic inflammatory remodeling and ecological depletion, ARDS exemplifies acute barrier collapse with systemic inflammatory amplification, and lung cancer represents a context of persistent inflammation, immune evasion, and therapy-modifying host–microbiome interactions. This approach enables a comparative synthesis of shared mechanisms while preserving important disease-specific differences. Framing them in this way allows the comparison of shared mechanisms across kingdoms in different pathological and biological contexts, while maintaining disease-specific heterogeneity and avoiding deterministic interpretations. It also helps explain why similar microbes and immune circuits may produce different phenotypic outcomes depending on the host's inflammatory state, tissue context, and disease stage.^2^ ^,^ ^8-10^

**Figure 1.**
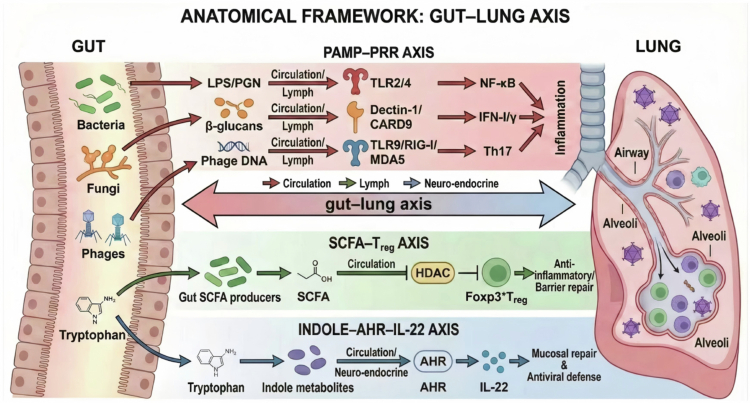
Integrated immunoregulatory network of the gut–lung axis. This schematic summarizes current concepts regarding how the gut microbiota and its metabolites remotely shape pulmonary immune homeostasis and inflammatory responses. Gut-derived microbe-associated molecular patterns (MAMPs), including lipopolysaccharide/peptidoglycan, *β*-glucans, and bacteriophage DNA, can access the lung via circulatory and lymphatic routes and are sensed by pattern recognition receptors such as TLR2/4, Dectin-1/CARD9, and TLR9/RIG-I/MDA5. The engagement of these receptors activates NF-κB signaling, interferon responses, and Th17-associated pathways, thereby promoting pro-inflammatory immune programs in the lung. In contrast, short-chain fatty acids (SCFAs) generated by gut microbial fermentation exert immunoregulatory effects by inhibiting histone deacetylases and promoting Foxp3⁺ regulatory T cell differentiation, contributing to the resolution of inflammation and maintenance of epithelial barrier integrity. In parallel, microbial metabolism of dietary tryptophan produces indole derivatives that activate the aryl hydrocarbon receptor–IL-22 axis, supporting mucosal repair and antiviral defense. Collectively, these interconnected pathways define a dynamic gut–lung immunological network with important implications for respiratory health and disease.

Therefore, this Review adopts an ecological and immunological perspective centered on cross-kingdom microbiome interactions. We summarize key interaction features among gut fungi, bacteria, and viruses. We then compare how these mechanisms are differentially manifested across asthma, COPD, ARDS, and lung cancer as non-sequential but partially intersecting disease contexts. Finally, we discuss the translational implications of this framework, including biomarker development and microbiome-targeted interventions. Our aim is to provide a theoretical basis for precision stratification and individualized treatment of respiratory diseases by “reconstructing the immune features of the gut–lung axis” (Figure 1).

## 2. Scope and literature search strategy

This Review is a narrative synthesis focused on cross-kingdom microbiome interactions along the gut–lung axis in respiratory disease. We searched PubMed, Web of Science, and Scopus for English-language studies published up to January 2026 using combinations of the terms “gut–lung axis”, “microbiome”, “mycobiome”, “virome”, “phageome”, “bacteriophage”, “fungi”, “*Candida*”, “SCFA”, “AHR”, “interferon”, “asthma”, “COPD”, “ARDS”, “sepsis-associated acute lung injury”, and “lung cancer”. We prioritized original human studies, mechanistic animal studies with clear immunological relevance, and recent high-quality reviews for background synthesis. Particular emphasis was placed on cross-kingdom interactions, barrier dysfunction, immune signaling, and translational implications. Because the available literature is heterogeneous in study design, disease focus, and methodological depth, this Review emphasizes comparative interpretation and critical appraisal rather than formal meta-analysis.

Because the available literature combines experimental studies, animal models, mechanistic inference, and human cohort observations, we apply an evidence-aware interpretive approach throughout this Review. The evidence is distinguished as experimentally demonstrated mechanisms, biologically plausible or hypothesis-generating models, and clinically supported human findings. Human cohort signatures, biomarker associations, and treatment-related observations are interpreted as clinically informative but not causal unless supported by interventional or rigorously causal evidence. This hierarchy is summarized in Table 1 and applied in the disease-specific sections below.

**Table 1.** Summary of the tri-axial signaling mechanisms underlying gut–lung immune crosstalk and their implications in respiratory diseases.

	Evidence hierarchy and interpretation								
Axis/Pathway	Experimental support	Biologically plausible/hypothesis-generating model	Clinically supported human findings	Major Microbial Source	Key molecules	Receptor/signaling pathway	Main effector cells	Functional Outcome on gut–lung axis	Representative diseases/scenarios
**PAMP–PRR**	Microbial PAMPs, including LPS/PGN, fungal *β*-glucans, and phage-derived nucleic acids, can activate TLR2/4, NOD-related pathways, Dectin-1/Syk/CARD9, TLR9, and interferon-related innate immune programs in defined experimental systems.	A plausible model is that gut barrier disruption or cross-kingdom dysbiosis increases exposure to bacterial, fungal, and phage-derived immune ligands, creating convergent PRR input to myeloid, epithelial, or endothelial compartments. This may lower the inflammatory threshold or amplify injury responses in distal lung disease contexts, particularly during acute injury or exacerbation. However, the complete gut-derived PAMP→ systemic immune activation→ lung disease progression pathway remains incompletely validated in humans.	Human studies have reported associations among circulating microbial products, microbial cfDNA, altered bacterial/fungal/phage signatures, inflammatory markers, disease severity, and outcomes in asthma, COPD, ARDS, and lung cancer; most findings remain associative rather than causal.	Bacteria, Fungi, Phages	LPS/PGN, *β*-glucans, phage DNA	TLR2/4, Dectin-1/CARD9, TLR9/RIG-I/MDA5 → NF-κB, IFN-I/γ, Th17	Macrophage, Th17 cells	Promote inflammation amplification, barrier disruption	Asthma exacerbation (Th17 amplification), COPD chronic inflammation, ARDS (strong activation triggered by "PAMP cocktail")
**SCFA–Treg**	SCFAs, especially acetate, propionate, and butyrate, can promote Foxp3 + Treg differentiation, anti-inflammatory signaling, epithelial barrier protection, and immune tolerance through mechanisms including HDAC inhibition and metabolite-sensitive immune regulation.	A plausible model is that loss of SCFA-producing bacteria reduces Treg-mediated immune restraint and epithelial barrier resilience, thereby creating a permissive inflammatory state that increases susceptibility to airway inflammation, infection-associated exacerbation, or acute lung injury. Whether restoring SCFA-producing communities or SCFA signaling can reproducibly improve human respiratory outcomes remains insufficiently validated.	Reduced SCFA-producing taxa, altered fecal SCFA levels, or SCFA-related microbial signatures have been associated with asthma phenotypes, COPD severity, critical illness/ARDS, and immune checkpoint inhibitor outcomes in some human cohorts, but these observations do not by themselves establish causal protection.	Gut SCFA-producing bacteria	SCFA (short-chain fatty acids)	HDAC inhibition → Foxp3⁺Treg	Treg cells	Antiviral, prevents excessive inflammation, anti-inflammatory/barrier repair	Acts as immune buffering in asthma; weakened or depleted in COPD and ARDS
**Indole–AHR–IL-22**	Microbial tryptophan metabolites can activate AHR signaling and promote IL-22-mediated epithelial repair, antimicrobial defense, mucosal barrier maintenance, and immune regulation in experimental systems.	A plausible model is that impaired microbial tryptophan metabolism weakens AHR–IL-22-dependent epithelial resilience and mucosal repair, thereby increasing vulnerability to barrier dysfunction, infection-driven inflammation, or delayed resolution of lung injury. This protective gut-derived indole–AHR–IL-22 model is biologically coherent but remains only partially validated in human gut–lung disease settings.	Human studies have reported altered tryptophan or indole-related metabolic signatures in asthma, COPD, critical illness, and inflammatory disease contexts, but direct clinical validation of a gut-derived indole–AHR–IL-22 causal pathway in respiratory disease remains limited.	The gut microbiota (metabolizing tryptophan)	Indole metabolites	AHR → IL-22	(Acts on epithelial cells via IL-22, etc.)	Barrier protection, mucosal repair & antiviral defense	Acts as protective "immune buffering" in asthma; axis is depleted in ARDS

## 3. The cross-kingdom gut microbiome as a systems regulator of the gut–lung axis

### 3.1. Intestinal mycobiome: fungal ecology and CARD9–Th17 signaling

Although fungi account for only approximately 0.1% of the total gut microbiome and show relatively unstable population dynamics, their importance in regulating mucosal immunity is increasingly recognized.^11^ ^,^ ^12^ Studies indicate that *Candida spp.* can activate the Dectin-1/Syk/CARD9 signaling pathway via cell-wall components, which induces dendritic cells to produce IL-6, IL-1β, and IL-23 and promotes Th17 differentiation. This response helps maintain antifungal homeostasis in the intestinal mucosa. CARD9 deficiency leads to fungal overgrowth and worsened mucosal inflammation.^13-17^ It is closely associated with increased susceptibility to inflammatory bowel disease (IBD). In addition, this “fungus–CARD9–Th17 axis” may influence lung inflammation through immune–cell trafficking, providing an important mechanistic basis for gut–lung immune crosstalk. These findings also suggest that it is an upstream regulatory component of gut–lung immune communication.^7^ ^,^ ^13^ ^,^ ^14^ ^,^ ^18^ ^,^ ^19^ In parallel, recent cell-tracing studies using Kaede photoconvertible mice and single-cell genomics have shown that colon-derived immunocytes can emigrate in an S1P-dependent manner and distribute to extraintestinal inflammatory and tumor sites, supporting immune-cell trafficking as a plausible mechanism of gut–distal organ communication.^20^

Clinical cohort evidence further supports the involvement of gut fungi in inflammation and carcinogenesis. In ulcerative colitis, patients in the active phase show a marked increase in *Candida* abundance, which decreases after remission. During remission, *Candida* abundance remains positively correlated with anti-inflammatory commensals, whereas this association disappears during active disease. This pattern suggests a close link between fungal dysbiosis and inflammatory activity. In colorectal cancer, the gut mycobiome also shows clear disruption, including enrichment of specific fungal taxa, breakdown of the original balance network, stronger fungus–fungus cooperation, and intensified bacteria–fungus antagonism. These findings indicate that the tumor-associated microbial ecosystem is restructured. Combinations of fungal features may serve as potential non-invasive biomarkers for screening and risk stratification.^21-25^

However, direct extrapolation from intestinal inflammatory or colorectal oncogenic settings to human lung cancer should be made cautiously, and in the present review, these observations are used primarily to support broader principles of fungal dysbiosis, immune activation, and ecological restructuring rather than direct gut-to-lung causality. Accordingly, intestinal fungal dysbiosis may not only reflect the gut inflammatory status, but may also be relevant to systemic immune modulation across respiratory diseases. However, the strength of evidence varies markedly by disease context, and direct causal support remains limited, particularly in lung cancer.

### 3.2. Intestinal virome and phageome: phage ecology and antiviral IFN circuits

In recent years, advances in metagenomics and virus-like particle (VLP) enrichment have greatly improved our ability to profile the human virome, drawing attention to the virome as an important component of the microbiome. Importantly, virome profiling is highly method-dependent. Bulk metagenomic sequencing and VLP-enriched sequencing provide complementary but non-identical views of the gut virome: the former may better capture prophage-associated sequences embedded within bacterial genomes, whereas the latter improves the detection of encapsidated viral particles and reduces the host/bacterial background. In addition, current human gut virome datasets remain biased toward DNA phages, whereas RNA phages are still comparatively underdetected and undercharacterized. This imbalance reflects technical limitations in terms of sample preservation, nucleic acid extraction, reverse transcription efficiency, library construction, reference database completeness, and annotation pipelines. Accordingly, the apparent predominance of DNA phages in published datasets should be interpreted cautiously rather than as a complete representation of the gut virome.^26-28^ The gastrointestinal tract is one of the most virus-rich ecosystems in the human body, and bacteriophages are the most abundant members.^29-34^ Phages regulate the gut ecosystem through lytic and lysogenic modes, mainly by shaping bacterial tolerance, metabolism, and competitive relationships within the community.^30^ ^,^ ^35^ Under homeostatic conditions, phages often form long-term coexistence relationships with dominant bacterial strains. This helps maintain microbial diversity and metabolic balance and generates an “ecological buffering” effect.^36^ ^,^ ^37^ However, during intestinal inflammation or dysbiosis, phages may expand and show a positive association with disease severity. This expansion can disrupt homeostasis and amplify inflammation, producing a “destabilizing” effect. Importantly, Gogokhia and colleagues showed that gut phages do not act only indirectly through changes in bacterial communities. Phage DNA can directly activate the TLR9–MyD88 pathway in dendritic cells, induce a Th1-type IFN-*γ* response, and thereby worsen intestinal mucosal inflammation.^6^

In addition, enteric viruses can be sensed by RIG-I/MDA5 in epithelial cells and antigen-presenting cells, which rapidly triggers an antiviral signaling program centered on type I interferons (IFN-I). In experimental and infection-related contexts, this response may contribute to a broader antiviral immune state. Through circulating cytokines, the migration of myeloid cells, and communication between the gut-associated and lung-associated mucosal immune systems, these signals could plausibly influence distal mucosal immune responsiveness to viral or bacterial challenges. Together, these observations support a conceptual and testable link between gut virome sensing and lung mucosal immunity: “gut virome–RIG-I/MDA5–IFN-I–lung mucosal immunity”.^38-42^

Recent evidence also supports lung-local phage mechanisms. Purified lytic *Pseudomonas aeruginosa* phages can interact with human cystic-fibrosis airway epithelial cells in a phage type- and pH-dependent manner and induce antiviral and proinflammatory mediators, including IFN-*β*, IFN-λ1, IL-8, TNFSF13B, and TNFSF8, with reporter assays implicating extracellular TLR1/2/4/6 activation. In COPD, respiratory metagenomic analysis further showed a progressive decline in bacteriophage diversity with increasing GOLD severity, disrupted viral–bacterial diversity coupling in frequent exacerbators, and an association between virulence factor-encoding *Haemophilus* phages and increased *Haemophilus* abundance. These findings suggest that the pulmonary phageome may be associated with epithelial PRR–cytokine responses and phage–bacterial community alterations, although direct in vivo evidence for gut-derived phages engaging these pathways in the human lung remains insufficient.^43^ ^,^ ^44^

Overall, the gut virome plays a central role in local ecological stability and inflammatory control. It can also transmit inflammatory or antiviral signals to the lung through two key immune pathways, TLR9–IFN-*γ* and RIG-I/MDA5–IFN-I. This framework helps explain the association between gut microbial shifts and susceptibility to respiratory diseases. Although direct evidence demonstrating identical phage-sensing pathways in airway epithelial cells remains limited, studies from gut-focused systems indicate that phage-derived nucleic acids can activate innate immune signaling and promote systemic inflammatory responses. Through the gut–lung axis, such systemic immune activation may provide a plausible explanation for the inflammatory associations observed in asthma exacerbations, COPD progression, and ARDS. Most direct evidence for these mechanisms is derived from intestinal or experimental systems rather than from direct studies in airway epithelial cells. In addition, while systemic dissemination of immune signals such as cytokine responses is biologically plausible, direct evidence linking gut phage sensing to specific circulating cytokine profiles or lymphocyte-mediated responses in human respiratory disease remains limited.

### 3.3. Cross-kingdom interactions within the gut microbiome

#### 3.3.1. Bacteria–fungi interactions: competition, cross-feeding, and PAMP–PRR synergy

Gut bacteria and fungi coexist within the same ecological niche and form dynamic cross-kingdom interaction networks.^45-48^

Their relationship includes both competition and cooperation. Competition occurs for nutrients and niche space, such as carbon sources, amino acids, and metal ions.^49-52^

Cooperation includes cross-feeding of metabolites and functional complementarity, which together shape the structure and stability of the gut ecosystem.^46^ ^,^ ^52-54^

Notably, fungal metabolites can directly reprogram bacterial metabolic phenotypes. For example, ethanol produced by fungi can act as a cross-kingdom signaling molecule. It can reshape bacterial metabolic states through regulatory pathways such as WspR/c-di-GMP and SpoT–AlgU, thereby promoting biofilm formation, mucoid phenotypes, and virulence-factor expression. This process does not require host involvement and represents a classic “metabolism-to-phenotype” cross-kingdom regulatory mechanism.^53^ Conversely, bacteria such as *Pseudomonas aeruginosa*, *Staphylococcus aureus*, and *Escherichia coli* can modulate fungal filamentation, biofilm formation, and virulence programs through quorum-sensing signals, metabolites such as phenazines and rhamnolipids, or cell-wall fragments such as peptidoglycan (PGN). Depending on nutrient availability and niche conditions, these effects can be bidirectional, either suppressive or promotive.^55-59^

Therefore, bacteria–fungi interactions are not passive coexistence. They are system-level ecological processes driven by nutrient competition, metabolic exchange, and signaling coordination. Their outcomes directly affect barrier homeostasis, mucosal immunity, and inflammatory susceptibility. This makes cross-kingdom ecological interactions a potential target for precise modulation of microbial stability and inflammation-related diseases.

#### 3.3.2. Bacteria–phage dynamics: kill-the-winner, piggyback-the-winner and horizontal gene transfer

Similar to fungi, bacteriophages are key cross-domain regulators in the gut ecosystem. Their interactions with bacteria have two major functions: predation control and horizontal gene transfer (HGT).^60^ First, lytic infection can constrain dominant bacterial populations and supports the classic “kill-the-winner” dynamic, thereby maintaining diversity and stability.^36^ ^,^ ^37^ ^,^ ^60-62^ Second, phages serve as major vehicles for HGT. During generalized and specialized transduction, phages can carry and transfer antibiotic resistance genes, metabolic pathway genes, and virulence-related genes to other strains or species. This accelerates bacterial adaptive evolution and reshapes community functions.^63-65^

Unlike the “kill-the-winner” pattern that is more apparent during periods of strong community fluctuation, phages under gut homeostasis often adopt lysogenic coexistence^37^ ^,^ ^66^ and the “piggyback-the-winner” (PtW) strategy.^26^ ^,^ ^67^ These modes allow phages to fine-tune bacterial abundance and competitive order without disrupting community structure, thereby supporting long-term stability and resilience.^31^ ^,^ ^66^ ^,^ ^68^

Multiple studies further show that phages can spread mobile genetic elements, including antibiotic resistance genes (ARGs), via specialized transduction, generalized transduction, and lateral transduction.^64^ ^,^ ^69-73^ Metabolic stress or inflammatory stimuli can markedly increase phage induction and gene-transfer events, making phages a major driver of resistance acquisition and dissemination in the gut. Therefore, phages not only shape bacterial community structure but also directly influence microbial evolution and drug-susceptibility patterns.^64^ ^,^ ^74-77^ In addition, the TLR9–MyD88–IFN-*γ* pathway described above suggests that phage–bacteria interactions are not only ecological processes, but also have meaningful immunological and systemic inflammatory consequences.^6^

Overall, bacteria–virus interactions in the gut integrate predation control, gene transfer, and immune regulation. They serve as a hub linking community stability, bacterial adaptive evolution, and host inflammatory responses. They also contribute to transitions between homeostasis and destabilization, and thus represent an important theoretical basis and potential intervention target for understanding gut–lung immune communication and susceptibility to respiratory diseases.

#### 3.3.3. Fungi–bacteria–phage triads and amplification of gut-derived immune signals

Recent studies increasingly suggest that gut fungi, bacteria, and the virome do not exist as independent modules. Instead, they form a cross-kingdom, multi-domain interaction network with dynamic feedback. A key emerging concept is the amplification interplay between fungal-derived signals in the gut and innate antiviral pathways triggered by respiratory viruses. This interplay provides an important entry point for understanding immune connections along the gut–lung axis.

On one side, fungal cell-wall components (such as *β*-glucan) can induce trained immunity through the Dectin-1–Syk/CARD9 pathway. This leads to metabolic and epigenetic reprogramming of myeloid cells, which increases the sensitivity of dendritic cells and macrophages to viral RNA sensing (the RIG-I/MDA5–MAVS axis). As a result, secondary viral stimulation can produce a substantially stronger IFN-I and interferon-stimulated gene (ISG) response.^78-80^

On the other side, respiratory viral infection can trigger systemic IFN-I responses through the RIG-I/MDA5–MAVS axis. These signals act not only in the lung, but can also reach the gut through circulating cytokines, the redistribution of myeloid cells, vagal reflex pathways, and metabolic regulation. They may alter intestinal mucosal immunity and microbial ecology. This pattern indicates that lung-derived antiviral signaling has systemic amplification properties.^81-84^

At the same time, the bacterial community is an active participant in immune and metabolic coupling between fungi and viruses. Bacterial metabolites such as short-chain fatty acids and lactate can regulate fungal *β*-glucan exposure and cell-wall signaling, suppress filamentation and biofilm formation, and thereby promote mucosal tolerance and ecological stability.^85-89^In contrast, bacterial pathogen-associated molecular patterns (PAMPs), such as lipopolysaccharide (LPS), can upregulate Dectin-1 expression on dendritic cells and enhance antifungal Th17 responses.^5^ ^,^ ^90^ Some studies also suggest that parallel Toll-like receptor (TLR) inputs can reshape the trajectory of *β*-glucan-induced trained immunity, leading to long-lasting pro-inflammatory memory.^91^ ^,^ ^92^ Thus, under dysbiosis or inflammatory conditions, synergy between bacterial PAMP signals and fungal PRR pathways can amplify ongoing inflammation and lower the threshold for future inflammatory responses, which may facilitate fungal pathogenic transitions. In addition, virus-induced IFN-I signaling, together with its metabolic and barrier effects, may alter bacterial niches and competitive structure in the gut.^93^ ^,^ ^94^ Respiratory viral infection can also alter the gut ecosystem through anorexia-related metabolic pathways.^95^

**Figure 2.**
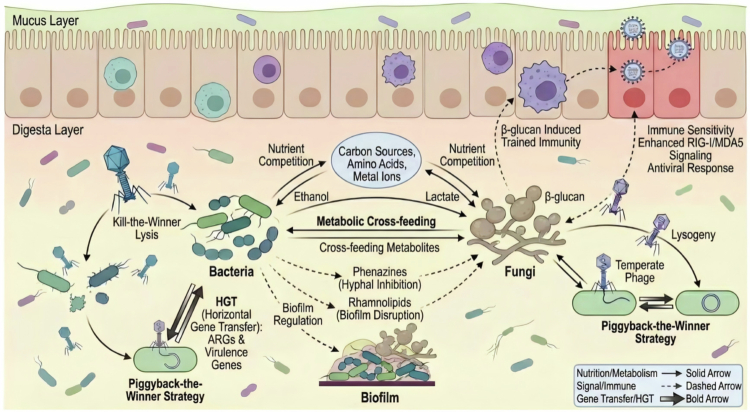
Local cross-kingdom dynamics in the gut. The gut ecosystem is governed by a tripartite interaction network involving bacteria, fungi, and bacteriophages. Bacteria and fungi engage in nutrient competition (e.g., for carbon sources and metal ions) and metabolic cross-feeding, where fungal-derived ethanol and bacterial-derived lactate act as inter-kingdom signaling molecules. Bacterial metabolites (e.g., phenazines) can inhibit fungal hyphal formation, while phage-mediated lysis (“Kill-the-Winner”) and lysogeny (“Piggyback-the-Winner”) regulate bacterial community structure and facilitate the horizontal gene transfer (HGT) of virulence genes. Notably, fungal cell wall *β*-glucans induce trained immunity in myeloid cells, enhancing the sensitivity of the RIG-I/MDA5 axis to viral challenges.

In summary, bacterial metabolites, PAMP–PRR signaling, and virus-driven interferon programs can be organized into a tri-axial interpretive framework for cross-kingdom microbiome interactions, rather than a fixed or universally validated mechanistic model. Within this framework, fungi influence whether immune responses are “amplified”, viruses influence the direction of amplification (antiviral defense versus inflammatory escalation), and bacteria influence the final outcome (maintenance of homeostasis versus destabilization). The gut–lung axis provides a biological context in which gut-derived and lung-local signals may interact. Therefore, local infections may alter the inflammatory state and susceptibility to secondary injuries, although the extent and direction of these effects still depend on the disease and specific circumstances. This tripartite interaction model offers a useful conceptual basis for comparing dynamic coupling along the gut–lung axis in infection, asthma, and COPD exacerbations, and selected cancer-associated immune contexts, although direct causal support varies substantially across diseases (Table 1, Figure 2).

## 4. Cross-kingdom gut dysbiosis along the gut–lung axis in respiratory diseases (Figure 3)

**Figure 3.**
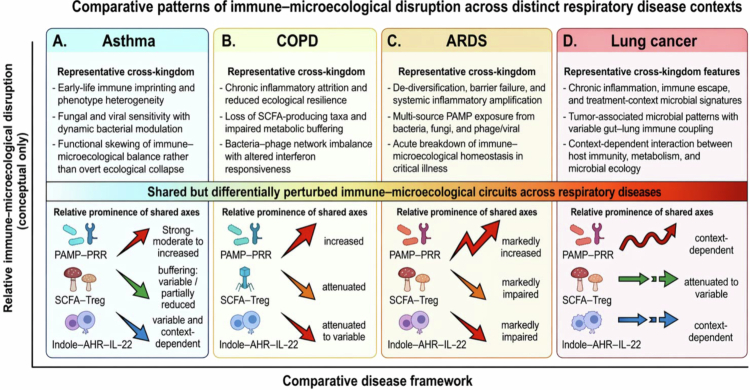
Comparative patterns of immune–microecological disruption across distinct respiratory disease contexts. This schematic presents a comparative and non-sequential framework for asthma, chronic obstructive pulmonary disease (COPD), acute respiratory distress syndrome (ARDS), and lung cancer, organized according to differing patterns of immune–microecological disruption rather than a deterministic disease sequence. Across these disease contexts, the relative contribution of PAMP–PRR signaling, SCFA–Treg buffering, and indole–AHR–IL-22 activity differs according to inflammatory intensity, barrier integrity, treatment exposure, and host background. Asthma is characterized by functional microbial shifts and early mycobiome alterations, with moderate activation of the PAMP–PRR axis and weakened immunoregulatory pathways. In COPD, chronic microbial depletion, reduced short-chain fatty acid (SCFA) production, and disruption of phage–bacteria networks result in further amplification of innate immune signaling. In ARDS, loss of microbial diversity, epithelial barrier breakdown, and a complex PAMP milieu drive extreme activation of PAMP–PRR signaling, while SCFA–Treg and indole–AHR axes collapse. In the context of the lung cancer setting, chronic inflammation, immune escape, and treatment-context microbiome interactions are particularly prominent. Overall, this model is intended to compare shared and distinct cross-kingdom features across respiratory diseases, not to imply a fixed temporal progression from one condition to another.

### 4.1. Asthma and allergic diseases

Asthma is a typical heterogeneous inflammatory airway disease. Its immune features are shaped not only by host genetics and environmental exposures, but also by bidirectional regulation between the gut and respiratory microecology. In a cohort study by Kei E. Fujimura and colleagues, neonates at approximately 1 month of age could be classified into distinct “microbial states” based on gut microbiome profiles. One high-risk state, defined as NGM3, was characterized by a marked reduction of protective bacteria, an abnormal fungal profile, and increased pro-inflammatory metabolites. The study showed that infants in the NGM3 group had a significantly higher risk of multi-sensitized atopy at 2 y of age and physician-diagnosed asthma at 4 y of age.^96^ Other reports also indicate that early-life shifts in the gut mycobiome are associated with childhood asthma and allergic susceptibility.^97^ ^,^ ^98^ Together, these findings suggest that early microecological states play a key role in immune remodeling and phenotype setting in asthma. Interpretation of microbiome findings in asthma should also consider important confounders, including age-dependent immune maturation, allergic background, dietary exposure, and the use of inhaled corticosteroids or biologic therapies, all of which may independently alter gut and airway microbial composition.

During acute asthma exacerbations, virus–fungus interactions are considered an important context for immune amplification. Respiratory viral infections such as RSV and influenza can perturb the intestinal microbial environment through systemic immune activation and infection-associated anorexia, and these host changes may create conditions permissive for gut mycobiota dysbiosis. The human H1N1 and COVID-19 cohorts further show increased gut fungal burden and altered fungal community structure. However, direct evidence that type I interferon-driven barrier or redox alterations are themselves responsible for fungal expansion in the gut remains limited.^6^ ^,^ ^95^ ^,^ ^99^ In this setting, *Candida*-associated fungal dysbiosis may add secondary innate immune inputs through TLR9- and Dectin-1-related signaling, promoting IL-6/IL-23-associated Th17-skewed inflammation.^7^ ^,^ ^13^ ^,^ ^14^ This cross-kingdom amplification can be summarized as a positive-feedback loop: “viral triggering → fungal expansion → secondary systemic inflammatory amplification”. It can worsen gut and lung inflammation in parallel. This mechanism helps explain why viral infections substantially increase the risk of asthma exacerbations in clinical settings.

In parallel, the immunoregulatory roles of gut microbe-derived indole metabolites are increasingly viewed as an important clue to asthma phenotype differences. Studies have shown that the gut microbiome, including bacteria such as *Lactobacillus* and some yeast-like fungi, can convert tryptophan into metabolites such as indole-3-aldehyde (IAld) and indole-3-acetic acid (IAA). These metabolites act through the aryl hydrocarbon receptor (AHR) to promote IL-22 production, which strengthens mucosal barrier function and antiviral defense.^100-103^ Therefore, the “microbial metabolism–AHR–IL-22” pathway may function as an immune buffer by promoting IL-22-dependent epithelial repair, mucosal barrier integrity, and antiviral defense, thereby limiting excessive inflammation. In asthma, this buffering capacity may help explain clinical heterogeneity: patients with weaker microbial indole production, AHR responsiveness, or IL-22-mediated repair may be more prone to progression under similar environmental or viral exposures.^104-106^

In addition, in asthma, bacterial communities not only shape immune outcomes at the system level, but also may exert bidirectional effects depending on metabolic status and the inflammatory milieu.^107^ ^,^ ^108^ On one side, as discussed above, SCFA-related immune buffering may counterbalance fungal- and virus-associated inflammatory amplification. In this setting, an SCFA-rich gut ecosystem may help preserve systemic immune tolerance, whereas reduced SCFA-producing capacity could lower the threshold for mixed Th2/Th17-skewed airway inflammation.^9^ ^,^ ^109^ ^,^ ^110^ On the other side, in inflammatory asthma contexts, bacterial PAMP signaling may reinforce fungal PRR-driven responses and thereby favor secondary inflammatory amplification.^5^ ^,^ ^90-92^

Overall, asthma-related interactions among fungi, viruses, and bacteria represent a dynamic balance process. The direction of imbalance determines whether the disease remains relatively stable or shifts toward acute exacerbation. In asthma, cross-kingdom disturbance can be described as a context in which fungal signals, viral triggers, and bacterial metabolic buffering interact dynamically, although the relative contribution of each component likely varies across endotypes and disease stages.

### 4.2. Gut–lung crosstalk in COPD: chronic low-grade inflammation and ecological collapse

COPD is a complex syndrome characterized by persistent airway inflammation and structural remodeling. Its pathogenesis is not restricted to the lung. It is closely linked to systemic chronic inflammation and multi-organ metabolic dysfunction.^111^ ^,^ ^112^ Recent studies indicate that gut–lung axis dysbiosis plays an increasingly important role in COPD onset and progression. Clinical cohorts and mechanistic work suggest that many COPD patients show increased gut permeability, impaired microbial metabolic functions, and elevated low-grade systemic inflammation. These changes can affect distal lung tissue through inflammatory mediators, microbial metabolites, and the migration of mucosal immune cells. Together, they form a gut–lung pathological loop in COPD.^10^ ^,^ ^113^ In COPD, interpretation of microbiome alterations is further complicated by smoking history, older age, sex imbalance across cohorts, recurrent antibiotic exposure, inhaled corticosteroid use, and nutritional status, all of which may independently shape bacterial, fungal, and viral communities.

In stable COPD, gut bacterial dysbiosis shows a relatively consistent pattern. Bowerman and colleagues used metagenomics and metabolomics to profile fecal samples from COPD patients. They found a significant reduction in SCFA-producing bacteria (such as *Faecalibacterium prausnitzii* and *Roseburia*), while potentially pro-inflammatory taxa such as *Streptococcus* were enriched. These shifts showed a continuous negative association with FEV₁%pred.^114^ This suggests that the loss of SCFA producers and anti-inflammatory metabolites, together with increased pro-inflammatory bacteria and abnormal metabolites, is correlated with impaired lung function and an increased inflammatory burden. The gut ecosystem is therefore not a passive bystander. It is linked to disease severity. Song and colleagues further summarized gut microbiome changes in COPD and proposed a shared trend: the gut ecosystem shifts from a “metabolic homeostasis type” toward a “pro-inflammatory vulnerable type”. This shift raises the systemic inflammatory baseline and reduces the immune compensatory capacity. It is more evident in smokers.^115^ Based on this, Wei Y. and colleagues provided preliminary causal evidence using Mendelian randomization. Genetic variation in certain gut microbial taxa (for example, *Actinobacteria*) was associated with COPD risk. This suggests that the gut microbiome may participate in susceptibility regulation rather than only reflecting disease status. It also supports the potential of gut microbes as biomarkers or risk predictors.^116^

Beyond bacteria, the virome (especially bacteriophages) is increasingly considered part of the gut–lung axis in COPD. Using viral particle enrichment sequencing, Liu and colleagues reported that the gut virome in COPD showed reduced diversity, altered *Caudoviricetes* phages, and weakened phage–bacteria interactions. Importantly, these changes correlated with systemic inflammatory markers such as CRP and IL-6, as well as lung function impairment. This finding indicates that COPD is not simply a state of “more phages”. It is better described as coordinated dysbiosis of the virome and bacteriome, which may weaken ecosystem resilience and immune homeostasis.^117^ These virome changes may be relevant to COPD not only because they reflect ecological instability (reduced viral diversity, altered Caudoviricetes phage profiles, and weakened phage–bacteria coupling), but also because altered phage–bacteria interactions could intersect with interferon-related immune disturbances and persistent inflammatory imbalance. In this context, virome/phage dysbiosis is better interpreted as part of a broader loss of ecosystem resilience and host–microbe immune coupling rather than as an isolated increase in phage burden. However, direct evidence demonstrating that cross-kingdom microbial alterations causally drive interferon dysregulation in human COPD remains limited, and current support is largely associative or derived from experimental inference rather than mechanistic proof in patients.^118-121^

At the functional level, COPD is characterized by loss of metabolic buffering rather than acute immune amplification. Clinical and review evidence suggests that reductions in SCFA-producing taxa, particularly *Faecalibacterium prausnitzii* and *Roseburia*, together with lower fecal SCFA levels, are associated with neutrophilic airway inflammation and greater disease severity.^113^ ^,^ ^115^ ^,^ ^122^ ^,^ ^123^ This pattern implies reduced tolerance to inflammatory stress and diminished metabolic support for barrier homeostasis. At the same time, enrichment of pro-inflammatory microbes and barrier dysfunction may facilitate the translocation of PAMPs such as LPS and peptidoglycan into the circulation, thereby contributing to low-grade systemic inflammation and increased susceptibility to excessive inflammatory responses during viral infection or environmental exposure.^10^ ^,^ ^124^

In summary, current evidence suggests three major features of gut–lung axis imbalance in COPD: (i) reduced SCFA producers and enrichment of pro-inflammatory taxa, which may weaken SCFA–HDAC–Foxp3⁺ Treg-mediated immune buffering and epithelial barrier support; (ii) reduced virome diversity and disrupted interaction networks (especially phage-related), which may accelerate niche shifts and functional instability; and (iii) combined effects of these microecological changes with systemic inflammation, oxidative stress, and immune aging, forming a cross-organ immune–metabolic network that shapes chronic progression and acute exacerbations. Although the causal direction needs further mechanistic validation, these findings provide a new ecological framework for the systemic nature of COPD and suggest that gut–lung axis–targeted strategies, including diet-based modulation, SCFA supplementation, microbiome restoration, and phage–ecology interventions, may hold therapeutic potential. Nevertheless, this therapeutic perspective remains provisional, as mechanistic support for SCFA-mediated immune buffering and phage–host immune interactions is still derived mainly from preclinical systems, whereas human COPD evidence is largely associative and cohort-based.

### 4.3. Gut barrier failure, PAMP “cocktails” and acute respiratory distress syndrome

Acute respiratory distress syndrome (ARDS) and sepsis-associated acute lung injury (S-ALI) are among the most typical critical-care phenotypes of “systemic inflammation and multi-organ interaction”. Traditional models focus on lung-local inflammation, endothelial injury, and disruption of the alveolar–capillary barrier. Increasing evidence now indicates that the gut is shifting from a “victim” to a “driver”. Severe infection, shock, hypoperfusion, mechanical ventilation, parenteral nutrition, and other factors can markedly damage the intestinal barrier. They can induce profound dysbiosis and promote leakage of bacteria, fungi, and their PAMPs into the circulation. This provides a sustained stimulus for systemic inflammatory storms and distal lung injury.^2^ ^,^ ^125^ ^,^ ^126^ Interpretation of microbiome signatures in this setting is further complicated by ICU-specific exposures, including broad-spectrum antibiotics, parenteral nutrition, vasopressors, sedation, and mechanical ventilation, all of which may independently reshape bacterial, fungal, and viral communities.

In ARDS and sepsis, many studies report a typical “de-diversification” pattern in the gut microbiome. Alpha diversity declines sharply. A small number of pathogen-enriched taxa, often including *Enterococcus*, *Staphylococcus*, or Enterobacteriaceae-family organisms, become dominant, while stool acetate, propionate, butyrate, and isobutyrate decline.^127-130^ As early as 2014, Zaborin and colleagues reported that in long-stay ICU patients, the gut community could be “reconstructed” into ultra-low-diversity ecosystems dominated by only 1–4 pathogens. These communities were often multidrug-resistant and strongly associated with infection and high mortality.^129^ More recent metagenomic and systematic-review evidence supports that loss of diversity and pathogen dominance are consistent across critical-care cohorts and correlate with systemic inflammation, nosocomial infections, and mortality risk.^131-133^

When de-diversification occurs together with barrier breakdown, large amounts of cross-kingdom microbe-associated molecular patterns can enter the bloodstream. This has been described as a “PAMP cocktail”. Multiple PAMPs can be detected in blood, including LPS, 1,3-*β*-glucan, and microbial cell-free DNA (bacterial/viral cfDNA). These signals reflect increased gut-derived microbial load and impaired barrier integrity.^134-136^ Persistent leakage of these PAMPs is associated with elevated inflammatory mediators, immune dysregulation, organ injury, and mortality risk. Notably, this is a multi-domain, multi-source, multi-pathway exposure pattern. This model differs from the classic single-pathogen infection model. When these PAMPs reach the lung, they may amplify ARDS-related injury by activating PRRs on macrophages/monocytes, neutrophils, endothelial cells, and epithelial cells, leading to NF-κB activation, NLRP3 inflammasome signaling, IL-1β/IL-6/TNF production, and increased alveolar–microvascular permeability. In ARDS, the key feature is not the emergence of entirely different mechanisms, but rather the synchrony, intensity, and loss of control of cross-kingdom signaling. Following barrier breakdown, mixed microbial signals from bacteria, fungi, and viruses/phages can converge on myeloid and endothelial compartments through overlapping PRR pathways, thereby amplifying cytokine release, inflammasome activity, and alveolar–microvascular barrier vulnerability.^131-139^ This multi-source “PAMP cocktail” distinguishes ARDS from more chronic respiratory disease contexts by creating a highly synchronized state of immune amplification (by generating simultaneous PRR activation, cytokine release, inflammasome signaling, and endothelial-myeloid activation) rather than more compartmentalized or partially buffered signaling.^134-136^ In parallel, early ARDS often shows collapse of protective metabolic programs, including reduced SCFA levels and impaired AHR-related epithelial repair.^125^ ^,^ ^127^ ^,^ ^140^ ^,^ ^141^ Experimental studies further suggest that exogenous butyrate or AHR agonists can partially improve barrier integrity and attenuate inflammatory injury.^140-142^ These findings suggest that metabolic-axis collapse may be a key turning point for uncontrolled cross-kingdom signaling in ARDS.

**Figure 4.**
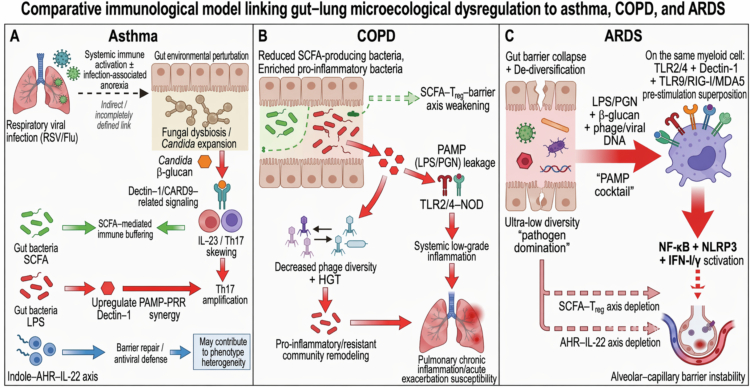
Comparative immunological model linking gut–lung microecological dysregulation to asthma, COPD, and ARDS. This model summarizes disease-context-specific mechanisms across distinct respiratory disease settings within a comparative gut–lung axis framework, without implying a linear or staged disease trajectory. (A) Asthma: Respiratory viral infections such as RSV and influenza may perturb the gut microecology through systemic immune activation and infection-associated anorexia, thereby creating permissive conditions for gut fungal dysbiosis, including Candida expansion. In this context, fungal *β*-glucans may engage Dectin-1–CARD9-related signaling and cooperate with gut-derived bacterial PAMP–PRR inputs to promote IL-6/IL-23-associated Th17-skewed inflammation. These pro-inflammatory effects may be partially counterbalanced by SCFA-mediated immune buffering and the microbial indole–AHR–IL-22 axis, which support mucosal barrier function and antiviral defense and may contribute to asthma phenotypic heterogeneity. (B) COPD: loss of SCFA-producing bacteria, enrichment of pro-inflammatory taxa, and reduced phage diversity increase gut permeability, allowing LPS/PGN translocation and activation of the TLR2/4–NOD pathways, driving systemic low-grade inflammation and chronic pulmonary injury. (C) ARDS: Barrier collapse and microbial de-diversification generate a PAMP cocktail that convergently activates PRRs on myeloid cells, triggering NF-κB, NLRP3, and interferon signaling, while regulatory SCFA–Treg and AHR–IL-22 axes are exhausted, culminating in alveolar–capillary barrier failure.

Overall, gut–lung axis imbalance in ARDS and S-ALI is distinguished by synchronized, high-intensity inflammatory amplification after barrier collapse. The disease-specific pattern is intestinal barrier failure → leakage of multi-source fungal/bacterial/viral PAMPs → convergent activation of myeloid and endothelial compartments → cytokine release, inflammasome activation, and alveolar–microvascular destabilization. This acute pattern differs from the more gradual ecological attrition observed in COPD patients and from the exacerbation-prone immune amplification observed in asthma patients. It remains an organizing model that requires direct validation in human critical illness (Figure 4).

### 4.4. Gut dysbiosis, fungal signatures, and lung cancer immune landscapes

Lung cancer, especially non-small cell lung cancer (NSCLC), is the leading cause of cancer-related mortality worldwide. Its development is considered the result of long-term interactions among genetic susceptibility, environmental exposures (such as smoking and air pollution), and persistent imbalance of the host immune microenvironment.^143^ Recent studies suggest that gut–lung axis dysbiosis is also an important contributor. Both lung-resident microbial communities and the gut microbiome have been implicated in processes relevant to lung carcinogenesis, including immune remodeling and treatment responsiveness, although the strength and causal depth of the evidence vary substantially by microbial domain and clinical context.^144-146^ Metagenomic and 16S rRNA sequencing data indicate that lung cancer patients often exhibit reduced gut alpha diversity, decreased SCFA-producing taxa (such as *Faecalibacterium* and *Roseburia*), and enrichment of pro-inflammatory genera (such as *Streptococcus* and *Prevotella*). These changes are associated with persistent activation of inflammatory mediators (IL-6, IL-8, and IL-17) and signaling pathways such as MAPK and inflammasome-related programs, which can support a chronic pro-tumor inflammatory environment in the lung.^146-148^ In addition, some gut microbes can drive systemic oxidative stress and immunosuppression through the LPS–TLR4–NF-κB axis, providing “remote support” for tumor initiation and progression.^147^ ^,^ ^148^ In lung cancer, microbiome interpretation is also subject to substantial confounding from age, smoking exposure, cachexia or malnutrition, proton pump inhibitor use, antibiotics, chemotherapy, radiotherapy, and immune checkpoint blockade, each of which may modify the gut and airway microbial ecology.

Here, the term “tumor-associated microbiota” is used in a broad sense to refer either to microbial signatures associated with a tumor-bearing host state or to microorganisms detected in proximity to tumor tissue. These two concepts should not be considered equivalent, because direct physical localization within tumors remains methodologically challenging in low-biomass tissues and may be influenced by contamination or sequencing artifacts. Even with these caveats, fungal signals derived from the gut and from the tumor-bearing host environment are increasingly attracting attention in lung cancer, particularly in relation to immune remodeling. Cohort studies reported that lung cancer patients have higher colonization rates and abundance of yeast-like fungi, such as *Candida,* in feces. In some individuals, the gut mycobiome is nearly “dominated” by a small number of fungi and is accompanied by coordinated shifts in specific bacterial genera, such as *Lactobacillus*. This suggests that the fungal–bacterial community structure is altered in patients with lung cancer, although a clearly defined cross-kingdom metabolic network has not yet been established in this disease context.^8^ ^,^ ^149^ Pan-cancer multi-omics analyses have further proposed the concept of the intratumor mycobiome. Different tumor types are enriched with different fungal genera. In lung cancer, *Blastomyces and Candida* are frequently reported, and their abundance is associated with tumor-related inflammation, immunosuppressive phenotypes, and prognosis.^150^ Some recent studies suggest that fungi detected in tumor-associated samples may be linked to immunosuppressive myeloid remodeling and tumor progression; however, these observations remain an active area of investigation and require careful interpretation in light of low-biomass sampling challenges and cross-study methodological heterogeneity.^151^ In parallel, studies have pointed out that in tumor models, migratory cells from the colon can accumulate at tumor sites, with myeloid cells being particularly prominent in a chemokine-dependent manner, suggesting that the migration of immune cells from the gut may impact the tumor immune environment. However, direct evidence of gut-to-lung tumor causality in humans remains limited.^20^ From a gut–lung axis view, gut *Candida* overgrowth and persistent fungal PAMP leakage could theoretically engage Dectin-1/CARD9-related Th17/IL-23 signaling and contribute to systemic inflammatory polarization with potential relevance to lung tumor immunity; however, direct gut-to-tumor causal evidence in humans remains limited.^8^ ^,^ ^149-151^ Thus, the mycobiome may contribute to tumor immune heterogeneity and variation in the immunotherapy response, but this interpretation remains incompletely validated.

Compared with bacteria and fungi, evidence on the lung cancer-associated virome and phage–bacteria interactions remains limited, but early signals suggest potential importance. On one side, long-term colonization and recurrent infections by respiratory viruses can shape lung mucosal immunity, and the virome composition in childhood and adulthood has been linked to chronic lung inflammation and tumor susceptibility.^152^ ^,^ ^153^ On the other side, several studies have shown that expansion and lineage restructuring of gut phages can reshape bacterial communities. Under antibiotic or inflammatory pressure, phages carrying resistance and virulence genes can be enriched. This can select for resistant or pro-inflammatory strains and sustain chronic inflammation.^63^ ^,^ ^75^In colorectal cancer, CRC-associated phage communities exhibit tightly coupled interaction networks with tumor-associated bacterial communities. These colorectal cancer findings support the possibility that phage–bacteria interactions may modify tumor-associated microbial ecology, but extrapolation to lung cancer remains speculative.^154^ ^,^ ^155^ These findings support the hypothesis that virome dysbiosis in lung cancer may influence tumor immunity by reshaping bacterial communities and metabolic profiles. However, this remains hypothesis-generating and requires validation in prospective gut–lung axis-focused cohorts and mechanistic studies.

Clinically, translational research on the gut–lung axis in lung cancer immunotherapy is particularly active. Multiple NSCLC and small cell lung cancer (SCLC) cohorts suggest that patients with higher gut microbial diversity, enrichment of SCFA-producing bacteria such as *Faecalibacterium*, and higher fecal SCFA levels achieve better response rates and survival benefits with immune checkpoint inhibitors (ICIs) targeting PD-1/PD-L1. In contrast, patients with long-term use of broad-spectrum antibiotics or proton pump inhibitors, or with marked dysbiosis, more often develop resistance to immunotherapy or increased immune-related toxicity.^156^ ^,^ ^157^ Some studies propose that SCFAs, especially propionate and butyrate, regulate dendritic cell phenotypes, CD8⁺ T-cell effector functions, and the Treg balance in a dose- and context-dependent manner. This finding supports the concept of SCFAs as “metabolic co-adjuvants” in antitumor immunity.^158^ Although evidence is still early, these observations support the hypothesis that cross-kingdom microbial and metabolic features may serve as translational biomarkers or adjunctive targets for improving immunotherapy responsiveness, rather than as established causal drivers of lung carcinogenesis.

Based on current evidence, the gut–lung axis in lung cancer can be summarized at three levels. First, tripartite interactions among bacteria, fungi, and viruses help shape chronic tumor-related inflammation and immunosuppression. Gut *Candida* expansion and intratumor fungi are emerging leads. Second, the PAMP–PRR axis and Th17/IFN pathways provide a conceptual framework for considering tumor-associated inflammatory polarization, but their direct role in human lung tumor initiation and progression remains incompletely validated. Third, microbial metabolic pathways, including SCFA- and indole/AHR-related signals, may participate in immune surveillance during carcinogenesis and in the modulation of immunotherapy response. Overall, lung cancer is better understood as an advanced disease context characterized by chronic inflammation, immune escape, and treatment-modifying microbiome interactions, rather than as a deterministic terminal endpoint of other respiratory disorders. Both lung-resident microbial communities and the gut microbiome have been associated with immune remodeling and therapeutic responsiveness in lung cancer, although the strength and causal depth of the evidence vary substantially by microbial domain and disease context. At the same time, interpretation of these microbiome signals requires caution because low-biomass sampling, contamination risk, treatment exposure, and cross-study methodological heterogeneity remain important limitations, particularly for fungal- and virome-related observations. Future progress will therefore depend on prospective gut–lung axis-focused cohorts and mechanistic studies that can more clearly define causal pathways and translational relevance.

## 5. Perspectives

Cross-kingdom microbial interactions within the gut–lung axis (bacteria–fungi–viruses) are moving from basic research towards clinically applicable biomarkers and therapeutic targets. Precision microecological interventions based on the proposed “tri-axial interpretive framework” (the PAMP–PRR axis, the SCFA–Treg axis, and the indole–AHR–IL-22 axis) may become an important component of future respiratory disease management. Potential strategies include: preserving intestinal barrier integrity and modulating PRR signaling to suppress pathological inflammatory amplification; restoring the protective metabolic axes of SCFAs and indole/AHR through dietary fiber, probiotics/synbiotics, or metabolite supplementation; and rebuilding cross-kingdom ecological networks using approaches such as phage therapy and multi-domain consortia that integrate bacteria, fungi, and phages. These directions may benefit not only chronic inflammatory diseases (asthma and COPD) and critical illness (ARDS), but also cancer immunotherapy, particularly PD-1/PD-L1 blockade, where they show potential to improve response rates and reduce toxicity.

Importantly, the gut–lung axis should not be interpreted as uniformly gut-centric. Across different respiratory diseases, the relative contribution of distal gut-derived immune and metabolic signals versus lung-resident microbial communities is likely to vary according to disease type, stage, treatment exposure, and host background. Future work should therefore better define when gut-centric processes dominate, when local airway ecological programs are more relevant, and how these distal and local microbial influences interact across different respiratory settings. Key priorities include establishing prospective cohorts, developing controllable models of gut–lung interactions, and defining the dose- and context-dependent effects of SCFAs and AHR signaling on immunotherapy. An integrated framework connecting cross-kingdom ecology, immunity, and metabolism suggests that respiratory disorders are being redefined from organ-limited diseases to systemic diseases. Within this framework, “reconstructing gut–lung axis immunity” may become a core direction for precision medicine.

A major challenge in interpreting gut–lung microbiome studies lies in the presence of substantial clinical and host-related confounders. Across different respiratory diseases, treatment regimens vary widely and may independently reshape bacterial, fungal, and viral communities, thereby complicating the attribution of observed microbial signatures to disease mechanisms alone. Moreover, age, sex, dietary patterns, smoking exposure, comorbidities, and disease severity can all influence microbial composition and function. These factors should therefore be taken into account when comparing studies, defining disease-associated microbiome patterns, and inferring mechanistic links along the gut–lung axis.

## 6. Conclusion

With rapid advances in cross-kingdom microbiology and mucosal immunology, the gut–lung axis has evolved from a descriptive association into a systems–biology framework with clearer causal and mechanistic foundations. Current evidence indicates that the cross-kingdom ecological network formed by gut bacteria, fungi, and the virome can influence pulmonary immune homeostasis through three core signaling axes: PAMP–PRR, SCFA–Treg, and indole–AHR–IL-22. Within this framework, respiratory diseases are better understood as distinct yet partially overlapping contexts of immune–microecological disruption. Asthma highlights early-life immune imprinting and phenotype heterogeneity; COPD reflects chronic inflammatory attrition and reduced ecological resilience; ARDS represents an acute state of barrier failure and multi-source inflammatory amplification; and lung cancer illustrates a more advanced disease context characterized by chronic inflammation, immune escape, and microbiome-linked treatment variability.

Although the strength and causal depth of the evidence remain uneven across microbial domains and disease settings, this cross-kingdom framework nonetheless points to several emerging translational directions. On this basis, reconstruction of cross-kingdom ecology, supplementation of protective metabolic axes (SCFAs and AHR ligands), phage–ecology modulation, combined fungus–bacteria interventions, and multi-domain microbial biomarkers (including PAMP exposure profiles and cross-kingdom signatures) all show substantial translational potential. Future work should prioritize mapping causal chains, integrating cross-kingdom multi-omics, developing controllable microbiome–immune models, and conducting clinical trials with microbiome-centered endpoints, in order to accelerate the development of truly precise therapies that modulate lung disease through the gut–lung axis by targeting distal gut-derived signals, local airway ecology, or their interaction in a context-dependent manner.
